# Blood Donation Practice among Undergraduate Students in a Tertiary Care Hospital: A Descriptive Cross-sectional Study

**DOI:** 10.31729/jnma.5288

**Published:** 2020-12-31

**Authors:** Pravakar Dawadi, Sabina Khadka, Milan Chandra Khanal, Raj Kumar Thapa

**Affiliations:** 1 Nepalese Army Institute of Health Sciences, Sanobharyang, Kathmandu, Nepal; 2 Shepherd College, New Baneshwor, Kathmandu, Nepal; 3 Department of Internal Medicine, Shree Birendra Hospital, Chhauni, Kathmandu, Nepal

**Keywords:** *blood donation*, *medical students*, *Nepal*, *transfusion*, *voluntary*

## Abstract

**Introduction:**

Voluntary blood donation is a reliable source of increasing the demand for blood transfusion. Medical students are the potential pool of blood donors. This study aims to find the prevalence of blood donation practice among medical students of a medical college in Nepal.

**Methods:**

This is a descriptive cross-sectional study conducted in a medical college of Nepal among students studying from the first year to final year MBBS. Ethical approval was obtained from the Institutional Review Committee of the Nepalese Army Institute of Health Sciences (Ref no. 245). A stratified random sampling technique was used to collect data. A self-administered pre-tested questionnaire was used to collect data. Data were analyzed using Microsoft Excel 2016.

**Results:**

The prevalence of blood donation practice among medical students of the medical college is 41 (22.20%) (17.35-27.05 at 95% Confidence Interval). The practice of blood donation is seen more among students of the final year 15 (35.71%) and the least among first year 3 (8.57%). Most of the donors, 24 (58.54%), have donated blood only once before. The most common reasons for donating and not donating blood before are ‘behavior of altruism’ 12 (29.27%) and ‘I am not fit/disapproved’ 44 (30.56%) respectively.

**Conclusions:**

This study shows less prevalence of blood donation practice among medical students. It points to the need for more extensive studies to explore the factors deterring medical students from donating blood. Definitive strategies are also needed to encourage medical students to increased voluntary participation in blood donation.

## INTRODUCTION

Blood transfusion is an essential aspect of health care, necessary for saving and improving the quality of life for millions of people around the world.^1^ The prevalence of blood donation in upper-middle-income and lower-middle-income countries is relatively low compared to the high-income countries.^2^

The scarcity of blood and blood products is commonly encountered in health care settings. Medical students can serve as a potential pool of blood donors in related teaching hospitals. Different studies have shown the poor practice of blood donation among medical students despite having relatively good knowledge and attitude towards voluntary blood donation.^3–6^

This study aims to find out the prevalence of the practice of blood donation among MBBS students studying from the first year to the final year of a medical college in Kathmandu, Nepal. Along with it, major motivations for blood donation, and the factors deterring medical students from donating blood is also studied.

## METHODS

A descriptive cross-sectional study was conducted in the Nepalese Army Institute of Health Sciences (NAIHS) among undergraduate students studying from MBBS first year to the final year. Ethical approval was obtained from the Institutional Review Committee of NAIHS Sciences (Ref no. 245) in June 2020. Students giving voluntary consent for data collection and available at the time of the study were included. Those who did not give voluntary consent and were not available during data collection were not included. The sample size was calculated using the formula as given below:

$$\begin{array}{ll} \text{n} & \text{= }{\text{Z}}^{\text{2}}\times \text{p}\times (\text{1}-\text{p)}/{\text{e}}^{\text{2}}\\ & \text{= }{\left(1.96\right)}^{\text{2}}\times 0.236\times 0.764/{(\text{0.05)}}^{\text{2}}\\ & \text{= }277.06\\ & =278 \end{array}$$

Where,

n = calculated sample size,
Z = 1.96 for Confidence Interval at 95%,
p = Prevalence from previous study, 23.6% 12
e = margin of error, 5%

In NAIHS, students currently studying from 1st to 5th year (N)= 537

Adjusted sample size= n/[1+{(n-1)/N]}]
= 278/[1+{(278-1)/537}]
= 278/1.52
= 182.89
= 183

Thus, the minimum number of the sample size required was calculated as 183. By adding 5% as a non-response rate, the sample size was taken as 192.

To incorporate students from all the five batches with an equal proportion of girls and boys from respective batches, we employed a stratified random sampling method. The list of students currently enrolled from MBBS first to final year was taken from the record section. A consecutive random number was assigned to the students on the list. Different sets of computer-generated random numbers were used to select the sample for each stratum. A total of ten sets of randomly generated numbers were used for each group of girls and boys in five batches. The number of samples to be selected from each of those groups was decided according to their proportion concerning the total population multiplied by the required sample size.

Data was collected using a pre-tested self-administered questionnaire with the help of an electronic form from July 1 to July 10, 2020. The questionnaire contained information related to gender, current year of study of MBBS, and whether they have donated blood before or not. Those who have donated blood before were further asked about the frequency of their blood donation till now, motivation regarding the blood donation, and if they donated blood after or before joining medical school. Those who have not donated blood before were further asked about the barrier behind not donating the blood before.

One student refused to give voluntary consent, and five responses could not be recorded as the students were not available during data collection. Similarly, one data was missing important information and was discarded as missing data. Thus, a total of 185 responses were taken into consideration.

The selection bias was minimized by random sampling, but biases like recall bias, confirmation bias, and information bias could occur.

The collected data was put into EXCEL 2016 and then edited and checked for consistency. After that, descriptive statistical analysis was done. The descriptive data was then presented as tables and pie charts.

## RESULTS

The point prevalence of the practice of blood donation among the students of MBBS first to final year in NAIHS was 41 (22.2%) [22.2±4.85 at 95% CI]. Among the 185 participants, there were 126 males and 59 females. Blood donation practice was found almost similar among male and female students. Out of 126 males, 28 (22.22%) and out of 59 females, 13 (22.03%) were found to have donated blood previously (Table 1).

**Table 1:** Blood donation history among MBBS students.

Gender	Total n	Donated Blood n (%)	
		Yes	No
Male	126	28 (22.22)	98 (77.77)
Female	59	13 (22.03)	46 (77.97)
Total	185	41 (22.16)	144 (77.84)

Among the students from five batches of MBBS, out of 41 blood donations made before, most of the blood donation was carried out by students from the fifth year 15 (35.71%), followed by the fourth year 10 (25.64%), the third year 7 (21.21%), the second year 6 (16.66%) and the first year 3 (8.57%) (Table 2).

**Table 2:** Distribution of previous blood donors among different years of MBBS students.

MBBS year	Total n	Previously donated n (%)
Fifth-year	42	15 (35.71)
Fourth-year	39	10 (25.64)
Third-year	33	7 (21.21)
Second-year	36	6 (16.66)
First-year	35	3 (8.57)
Total	185	41 (22.16)

**Figure 1:**
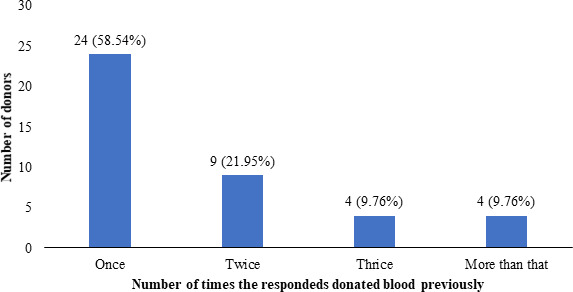
Frequency of blood donation practice among blood donors (n=41).

Out of the 41 students who have donated blood before, the number of times they have donated before was once 24 (58.54%), twice 9 (21.95%), thrice 4 (9.76%), and more than that 4 (9.76%) (Figure 1).

**Figure 2:**
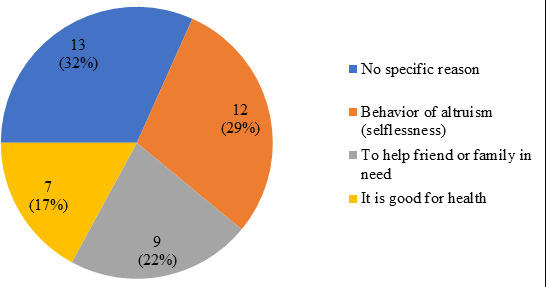
Motivations behind donating blood before among the donors (n=41).

The most common motivation behind donating the blood was ‘No specific reason’ in 13 (31.71%), followed by ‘Behavior of altruism’ in 12 (29.27%), ‘To help friend or family in need’ in 9 (21.95%) and ‘It is good for health’ in 7 (17.07%) (Figure 2).

Twenty-seven (68.85%) donors had donated blood after joining the medical school, whereas 14 (34.15%) donors donated before joining the medical school.

**Figure 3:**
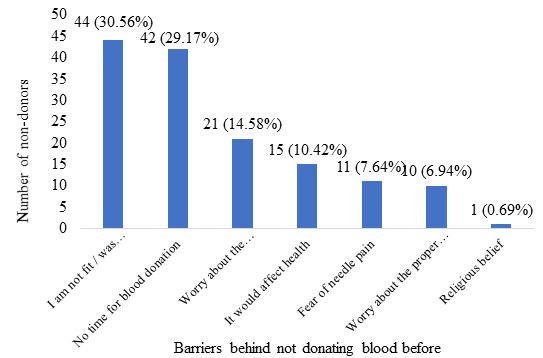
Barriers behind not donating blood before among non-donors (n=144).

The barriers behind not donating blood before among non-donors were ‘I am not fit/was disapproved for blood donation’ in 44 (30.56%), ‘No time for blood donation’ in 42 (29.17%), ‘Worry about the sanitation and getting infectious disease’ in 21 (14.58%), ‘It would affect health’ in 15 (10.42%), ‘Fear of needle pain’ in 11 (7.64%), ‘Worry about the proper use of donation’ in 10 (6.94%) and ‘Religious belief’ in 1 (0.69%) (Figure 3).

## DISCUSSION

The prevalence of students who had ever donated blood before in our study is found to be 22.2% which is relatively low. The study population is the medical students, who are assumed to have relatively higher knowledge and positive attitude toward voluntary blood donation. However, this knowledge does not seem to be translated into actual practice as per our findings. A study by Gomes M. J. et al. reports only a 12.7% prevalence of blood donation history among medical students.^3^ Similarly, another study by Tariq S. et al. also shows only 10.7% of students donated blood before.^4^ Similar kind of trend can be observed in the findings of other another by Melku M. et al.^5^ A study conducted in India reports that despite having relatively good knowledge and attitude about voluntary blood donation among medical students, the prevalence is very low as 22.9%.^6^ As the whole world races to find the appropriate blood group blood for transfusion in case of medical interventions, the same is the case with health care facilities here in Nepal. The barriers faced by the potential pool of those donors need to be explored and addressed to increase the voluntary blood donor counts.

A study conducted by Ugwu N. I. et al. in Nigeria among medical students reports that 69.6% of blood donors have only donated once, and 16% donated twice before.^7^ Also, in another study, there were only 13.6% of students as regular blood donors.^8^ Tariq S. et al. also reported that 8.7% of donors are one time donors among medical students. They only found that 1.5% of students have donated blood twice before, and 0.4% of donors as regular donors.^4^ Therefore, studies have shown that a large proportion of blood donors among medical students tend to donate blood only once. The same kind of trend has been reported by Majdabadi H. A. et al.^9^ and Chauhan R. et al. A small number of those donors have been found to donate blood more than once, and a tiny proportion of those are found to be regular donors. Our study also reports similar findings stating that 58.54% of donors are only one time donors. Not only in medical students, but this trend can also be seen in other students as well as reported by Suen L. K. P. et al.^10^ where there were only 22.39% of the donors among university students were regular donors. This tendency among blood donors has to be studied extensively to find what could be deterring them from donating again. Along with the new donor population, and already existing one-time donor population can be a potential source of future blood donation pool. So, the underlying cause or lack of motivation among those pool of people needs to be studied in further studies.

The study by Suen L. K. P. et al. reports more females among the blood donor's pool. There were 63.1% female and 36.9% male blood donors of Hongkong.^10^ However, in our study, the proportion of blood donors has been found almost the same between males and females, i.e., 22.22% and 22.03%. It is highly affected by our stratified random sampling method as there was an equal proportion of students recruited from males and females of every batch. Regardless, the study by Muhammad Y. implicates very few proportions of females were donating blood than men, i.e., 99.7% were male donors, and 0.3% were female donors among 10,799 blood donors.^11^ Although there were very few female donors, the number of recipients was not found to be similar in the same study. There were 56% females and 44% males among the blood recipient population. This study conducted in Nigeria highly suggests that there is a need to provide education and awareness regarding blood donation among the female population to correct the potential misconceptions or discrimination to meet up the increasing demand for blood. Similarly, another study by Majdabadi H. A. et al. reports there was a significant relationship between gender and awareness for the history of blood donation stating there were 2.26% female and 7.47% male blood donors among the study population.^9^

Our study population had an equal proportion of participants from all the five batches of MBBS students. We found out that most of the students having a history of blood donation, 35.71% were from the final or fifth year of MBBS, while the least of the donors, 8.57%, were from the first-year MBBS. First-year MBBS students are generally unexposed to the clinical environment as they only deal with basic science in the initial two years. Fifth-year students being most exposed to the clinical environment generally have more knowledge and a positive attitude towards the importance and necessity of voluntary blood donation in the clinical setting. Similar findings were reported by Tariq S. et al. where 26% of the donors were studying in the final year, 22% in the fourth year, 30% in the third year, 14% in the second year, and 8% in the first year.^4^ Another study conducted among the third year and fifth-year medical students concluded that the knowledge and willingness to donate blood were considerably greater among the fifth year students.^8^ To penetrate the junior year medical student population, which can be a promising pool of blood donors, appropriate knowledge and awareness regarding the significance of transfusion should be disseminated by the college itself or by the senior students. The medical colleges can also arrange facilities for exposure of those junior medical students in the clinical environment once in a while.

Our study indicated the major motivation for donating blood before were due to the moral responsibility of altruism 29%, and 17% thought it was good for health. It is common for people to be selfless when it comes to saving other people's lives, especially when it comes to those people who are already associated with a health science background. A study by Gao L. and QWang among students of continuing medical education reported a majority of 98.58% of the donors mentioned the reason behind donating blood before as altruism.^12^ Similar kind of finding was suggested by a review article related to knowledge, attitude, and practice surveys in developing countries by Lownik E. et al. It indicates that the most common motivating factor in all of the surveys was the appeal to altruism.^13^ The feeling of altruism is, by default, commonly found in most of the medical students as they have decided to devote their life to serving others while entering into the medical school. However, this is not by rule present in all of the students. This feeling of selflessness can arise among other students as well if the significance of blood donation in a health care facility can be shown to them in person during routine clinical posting and hospital visits from the early stage.

Most of the students, 36% in our study reported that they were not fit/disapproved for the blood donation before. The similar thought of the non-donor population was found out by Javaeed A. et al. among undergraduate medical students.^14^ The study stated that 42.1% of non-donor students thought they were not fit for blood donation. Most of the students do not have proper knowledge regarding the criteria necessary to be eligible for drawing out blood during blood donation, although they are medical students. This happens mostly in students of preclinical years. A similar trend was seen in another study by Melku M. et al. as 24.3% of the non-donor undergraduate health science, students thought they were medically unfit for blood donation.^5^ Proper awareness regarding the eligibility of blood donation before conducting the blood donation program could potentially motivate the eligible donors who have not donated blood, thinking that they are not eligible. There could be some underlying condition or lack of physical fitness that could have been deterring them from donating blood. Raghuwanshi B. et al. reported that anemia was the most common reason behind the non-donor population to be medically unfit for blood donation.^15^ So, there is a need for proper study for the determinants behind the inability of motivated blood donors to contribute their blood to the blood bank.

Medical school is one of the very challenging places concerning the devotion of time one has to give for passing out. Therefore, medical students tend not to have ample time for other activities beyond their studies and regular activities. Among the students who have not donated blood before in our study, 29.17% pointed to lack of time as the second most barrier for not donating blood. Less proportion of non-donor 8.82% blamed lack of time for being the cause for not donating blood before according to Raghuwanshi B. et al.^15^ Whereas, the insufficiency of time was regarded as the cause of deterring against donating blood by more proportion of non-donors in other studies by Taş A. and E. D. EvciKiraz 37.39%,^8^ Tariq S. et al. 36.2%^4^ and Dean B. W. et al. 36.8%.^16^ This problem could be addressed by organizing the blood donation camp inside the medical college or corresponding hospital itself, which would not take away much of the time from the busy schedule of medical students.

In our study, 14.58% of non-donor participants were worried about sanitation and getting infectious diseases. A study by M'Baya B. et al. shows the prevalence of transmissible transfusion infections like HIV, HBV, HCV, and syphilis among voluntary blood donors.^17^ Another study by Chen, J. T. et al. reports HIV infection in 260 cases out of 1,461,129 donors in Hangzhou.^18^ Other studies also reported the risk of infection and worrying about sanitation as the reason given by non-donors for not donating blood before.^19,20^ As health science students are relatively more aware regarding the safety; they are often found to have test seeking behavior during blood donation for screeing.^21^ The volunteers need to explain the safety measures adequately to protect donors from transfusion-related infections. They also need to ensure the ultimate sanitation in the area of donation, which will help the interested donors to proceed withthe donation.

Some students 3.1% are also found to be worrying about the proper use of their donated blood according to the study by Javaeed A. et al.^14^ Similarly, 7.6% of medical students who have not donated blood before were worried regarding the misuse of the blood in hospitals as reported by Ugwu N. I. et al.^7^ In our study also, 6.94% of non-donors stated they were worried about the proper use of the donated blood. It shows the lack of trust of the students over the agencies and organizations who are collecting their blood. This misunderstanding could be addressed by informing the students about the process involved between taking their blood and giving the blood to a recipient. Once they get a hold of the overall process, and they have trusted that the recipient would get the necessary blood without much of a hassle, they will certainly be ready to donate the blood.

Our study showed only 0.69% student out of all non-donors referred to religion as the barrier for not donating blood previously. A study by Zucoloto M. L., et al. reports a low association of religion with students who never donated blood before.^22^ Whereas, another study by Gebresilase H. W. et al. reports religion to be one of the significant predictors of knowledge regarding blood donation among students.^23^ Similarly, 6% and 7.6% of non-donors indicated religion and cultural belief as to the barrier for not donating blood before, as reported by Javaeed A. et al.^14^, and Ugwu N. I. et al.^7^ respectively. Our study population consists of the Hindu population mostly, and there were no reported restrictions imposed by the Hindu community over blood donation practice. This factor could alter on a regional basis, but it may be improved over appropriate knowledge dissemination among the people other than the ones restricted by their cultural beliefs.

The sample from our study was only taken from one medical college. So, the findings cannot be generalized to other medical colleges as well as students from other faculties. The reliability of our data is entirely based upon the correct reporting of the participants. So, recall bias might occur. Also, only the most common motivation and barriers towards blood donation, as suggested by the pre-test, have been studied. An in-depth qualitative study is necessary for the extensive study of those factors.

## CONCLUSIONS

The continuing demand for blood transfusion has to be met by voluntary donation. The future health professionals and present-day medical students can create a significant impact on this by donating blood themselves as well as motivating others. However, the less practice of blood donation among medical students in our study shows there is a need for more studies to explore the barriers faced by them, and it should be addressed accordingly. Furthermore, the frequency of blood donation can be increased by understanding the motivations behind the donors to donate blood again and again. Strategies necessary for encouraging medical students towards increased participation in voluntary blood donation should be designed
